# Simultaneous Cranioplasty and Subdural-Peritoneal Shunting for Contralateral Symptomatic Subdural Hygroma following Decompressive Craniectomy

**DOI:** 10.1155/2015/518494

**Published:** 2015-03-23

**Authors:** Muh-Shi Lin, Tzu-Hsuan Chen, Woon-Man Kung, Shuo-Tsung Chen

**Affiliations:** ^1^Department of Surgery, Faculty of Medicine, School of Medicine, National Yang-Ming University, Taipei, Taiwan; ^2^Department of Neurosurgery, Taipei City Hospital, Zhong Xiao Branch, Taipei, Taiwan; ^3^Department of Biotechnology and Animal Science, College of Bioresources, National Ilan University, Yilan, Taiwan; ^4^Department of Physical Medicine and Rehabilitation, Shin Kong Wu Ho-Su Memorial Hospital, Taipei, Taiwan; ^5^Department of Exercise and Health Promotion, College of Education, Chinese Culture University, Taipei, Taiwan; ^6^Institute of Biomedical Engineering, College of Medicine and College of Engineering, National Taiwan University, Taipei, Taiwan; ^7^Department of Neurosurgery, Lo-Hsu Foundation, Lotung Poh-Ai Hospital, Luodong, Yilan, Taiwan; ^8^Department of Mathematics, Tunghai University, Taichung, Taiwan; ^9^Sustainability Research Center, Tunghai University, Taichung 40704, Taiwan

## Abstract

*Background.* Contralateral subdural hygroma caused by decompressive craniectomy tends to combine with external cerebral herniation, causing neurological deficits. *Material and Methods.* Nine patients who underwent one-stage, simultaneous cranioplasty and contralateral subdural-peritoneal shunting were included in this study. Clinical outcome was assessed by Glasgow Outcome Scale as well as Glasgow Coma Scale, muscle power scoring system, and complications. *Results.* Postoperative computed tomography scans demonstrated completely resolved subdural hygroma and reversed midline shifts, indicating excellent outcome. Among these 9 patients, 4 patients (44%) had improved GOS following the proposed surgery. Four out of 4 patients with lethargy became alert and orientated following surgical intervention. Muscle strength improved significantly 5 months after surgery in 7 out of 7 patients with weakness. Two out of 9 patients presented with drowsiness due to hydrocephalus at an average time of 65 days after surgery. Double gradient shunting is useful to eliminate the respective hydrocephalus and contralateral subdural hygroma. *Conclusion.* The described surgical technique is effective in treating symptomatic contralateral subdural hygroma following decompressive craniectomy and is associated with an excellent structural and functional outcome. However, subdural-peritoneal shunting plus cranioplasty thoroughly resolves the subdural hygroma collection, which might deteriorate the cerebrospinal fluid circulation, leading to hydrocephalus.

## 1. Introduction

A wide decompressive craniectomy (DC) is indicated for conditions such as increased intracranial pressure (ICP) or diffused cerebral edema caused by major traumatic brain injury (TBI) and ischemic or hemorrhagic stroke [1–4]. DC involves removal of a large bone flap on the side of the affected brain hemisphere and simultaneous dural opening to create more space for the swelling brain. DC is considered as a technically simple surgery improving long-term outcomes with low incidence of complications [1–7]. However, impeded absorption of the cerebrospinal fluid (CSF) circulation leading to subdural hygroma (SDG) is a possible consequence of DC [8–10].

The exact incidence of SDG following DC remains unknown. In patients with TBI who have undergone a DC, the reported incidence of SDG varies from 6.5 to 57.4% [11, 12]. Specifically, the development of contralateral SDG after DC has been reported in very few studies [12–15], and this is believed to be an infrequent complication of DC [15]. In DC-treated patients, the incidence rate of ipsilateral SDGs has been reported to be significantly higher than that of contralateral SDGs [12]. A large-scale study of 39 SDGs by Aarabi et al. showed notable statistical significant difference in postoperative incidence rate between the ipsilateral and the contralateral ones (92% and 8%) [12]. In addition, contralateral SDG is usually combined with external cerebral herniation, causing irreversible neurological deficit. The occurrence rates of SDGs with mass effect for the ipsilateral and the contralateral ones were significantly different (2.8% and 66.7%, resp.) [12]. The underlying reason for a high tendency to global neurological decline has not yet been fully elucidated.

Currently, there are various ways to treat SDG, which include conservative treatment, subdural tapping, burr hole drainage, subdural drainage, or placement of a subdural-pleural or subdural-peritoneal (SP) shunt [9, 12, 16, 17]. However, any novel treatment that has fewer complications and allows significant functional recovery would be an important therapeutic advancement for patients with neurological deficits caused by contralateral SDGs.

Here, we describe a technique involving one-stage, simultaneous cranioplasty to reconstruct the skull defect and contralateral SP shunting to effectively drain the contralateral SDG in 9 TBI patients with contralateral SDG after DC. In this paper, we present the cases, treatments, and outcomes along with an overview of the pathogenesis and neurophysiology.

## 2. Materials and Methods

### 2.1. Ethics Statement

All patients provided their written informed consent and all procedures were approved by the ethics committees of Taipei City Hospital in accordance with Declaration of Helsinki.

### 2.2. Patients

Nine patients (6 males and 3 females, mean age: 46.2 ± 15.5 years, and age range: 31–71 years) having contralateral SDG following DC for TBI in our institute, between January 2008 and July 2011, were included in the study. Contralateral SDG was defined as a new-onset SDG on the side opposite to DC having computed tomography (CT) density identical to that of CSF.

**Figure 1:**
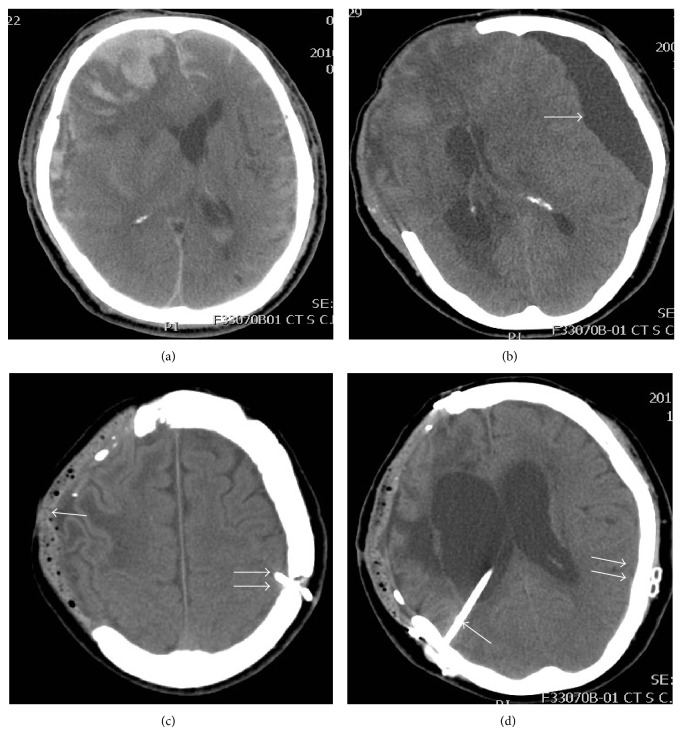
Representative pre- and postoperative CT images of case 1. (a) CT scan of case 1, revealing acute subdural hematoma, contusion hemorrhage, and subarachnoid hemorrhage in the right frontotemporal region, with significant mass effect. Wide craniectomy with the removal of hematoma was indicated. (b) A later follow-up CT scan revealed SDG contralateral to DC (arrow), with significant mass effect. (c) Simultaneous cranioplasty (single arrow) and contralateral SP shunting (double arrow) were performed and postoperative CT scan revealed completely resolved SDG and reversed midline shift. (d) A later follow-up CT scan revealed hydrocephalus and VP shunt (single arrow) following SP shunt (double arrow) insertion earlier to eliminate both hydrocephalus and contralateral SDG, respectively.

All the patients had severe head injuries and intracranial hemorrhage (Figure 1(a)) with marked neurological deficits and underwent cranial decompressive surgery. The type of lesions in the patients included acute subdural hematoma and massive intracerebral hemorrhage. The surgical methods included wide craniectomy with the removal of the hematoma.

At an average time of 21 days after craniectomy, all the patients presented with symptomatic contralateral SDG (arrow in Figure 1(b)) and underwent surgical intervention of simultaneous cranioplasty and contralateral SP shunting. Surgical indications for symptomatic contralateral SDG were the presence of persistent neurological deficits related to the hygroma, when follow-up CT scans revealed that the thickest diameter of the lesion was more than 7 mm [18] and a shift in the midline structures was more than 5 mm [12].

### 2.3. Operative Technique of Contralateral Subdural-Peritoneal Shunting

The procedure for SP shunt insertion was similar to our previous work on ventriculoperitoneal (VP) shunt surgery [19]. In brief, a burr hole was drilled on the skull at the dependent part of the accumulated SDG. The subdural space was opened and a cranial subdural catheter (2.5 mm in inner diameter and 4.0 mm in outer diameter) was inserted. A subcutaneous tunnel was made with a catheter passer from abdomen to the cranial burr hole. The tubing used at the distal end was the distal section of a VP shunt, which was valveless with an open end. The subdural catheter was connected to the abdominal catheter. The length of the intraperitoneal component of the catheter was measured to be approximately 35 cm when the operation was finished.

### 2.4. Cranioplasty for Skull Defect

We used polymethyl methacrylate (PMMA) or three-dimensional (3D) titanium mesh to reconstruct the skull defect. The surgical procedures have been described in previous studies [20, 21].

### 2.5. Evaluation and Follow-Up of Neurological Function

Clinical outcome was evaluated before and after surgery according to the Glasgow Outcome Scale (GOS) [22]. The Glasgow Coma Scale (GCS) and the muscle power scoring system were used to evaluate neurological condition, as described in our previous works [23, 24].

### 2.6. Statistical Analysis

Continuous data are shown as the mean ± standard deviation (SD), while categorical data are represented by numbers (*n*) and percentage (%). The descriptive statistics were performed using SPSS 15.0 statistical software (SPSS Inc., Chicago, IL, USA).

## 3. Results

### 3.1. Patient Characteristics

All the patients of the current study had symptomatic contralateral SDG following DC which was managed with simultaneous cranioplasty (single arrow in Figure 1(c)) and contralateral SP shunting (double arrow in Figures 1(c) and 1(d)). The mean time interval between DC and the development of contralateral SDG was 20.9 ± 3.4 days (range: 15–26 days). The major symptoms of contralateral SDG included decreased muscle power on both arms and legs localized to one side (*n* = 7) and lethargy (*n* = 4). The average midline shift on CT scan of these patients was 7.78 ± 4.47 mm (range: 3–15 mm). The cranioplasty materials for the patients were either PMMA (*n* = 8) or 3D titanium mesh (*n* = 1). SP shunting for SDG was performed during the same operation. Using this method, the mean surgical time was 251.3 ± 55.29 minutes (range: 150 to 314 minutes). The mean duration of follow-up was 32.67 ± 14.88 months (range: 10–51 months).

### 3.2. Postoperative Radiological Evaluation

Postoperative CT scans of all the patients revealed completely resolved SDGs and reversed midline shifts, indicating excellent outcome.

### 3.3. Postoperative Neurological and Long-Term Outcome Evaluation

Preoperative GOS scores of the 9 patients before the described surgical technique were 3, 3, 4, 3, 3, 2, 2, 3, and 3. After a mean follow-up of 32 months, the respective GOS scores of these patients were 3, 3, 5, 4, 4, 2, 2, 3, and 4 after surgery. Among theses 9 patients, 4 patients (44%), with preoperative GOS of 4, 3, 3, and 3 and postoperative GOS of 5, 4, 4, and 4, respectively, had improved GOS score while 5 patients' scores remained unchanged in GOS after simultaneous cranioplasty and contralateral SP shunting.

In detailed postoperative neurological assessment, within one week after surgery, the patients with the symptom of lethargy (*n* = 4) became alert and orientated. The muscle strength improved significantly 2 months after the surgery in 6 out of 7 patients and it remained unchanged in one patient even after 3 months of surgery. However, the patient had improvement of muscle strength after 5 months with aggressive rehabilitation therapy.

### 3.4. Complications

Two out of 9 patients (22.2%) presented with drowsiness and general weakness at an average time of 65 days after surgery. Their head CT revealed hydrocephalus and the time intervals between the initial DC surgeries to the hydrocephalus were 55 and 75 days. These patients underwent a VP shunt surgery (single arrow in Figure 1(d)) and regained consciousness within one week after the surgery. The ventricles were reduced in size as evidenced by the head CT after two months of the surgery.

To date, there have been no further recurrences of SDGs or hydrocephalus in any of these patients. Furthermore, there have been no cranioplasty implant extrusions or intracranial complications such as persistent headaches, meningitis, osteomyelitis, brain abscess, CSF leaks, pneumocephalus, or single/double gradient shunt malfunctions in any of these patients.

## 4. Discussion

In the present study, we introduce a surgical method of simultaneous cranioplasty and contralateral SP shunting in TBI patients with contralateral SDG after DC. Our data indicates excellent outcomes in terms of remarkable radiological and clinical improvements and very few complications. Although only 44% of patients had improved GOS score after a long-term follow-up of 32 months after surgery, the outcomes in GOS are amenable, given the severity of the initial injury necessitating a DC, and the occurrence of SDG-related complications following DC. Specifically, neurological evidence proved that all patients had improvement either in consciousness or limb weakness following our proposed method. Though unitary cranioplasty and SP shunting technique are common methods, the patients were managed well by a series of new surgical combination in our study which may be worth learning for surgeons. Our results meet the proposed aims of design to effectively treat contralateral SDGs, which may relate to postoperative neurological deterioration or increased ICP [12, 17].

SDG is defined as an acute or a chronic accumulation of CSF in the subdural space, the composition of which is frequently varied [9]. The natural history of SDG after DC is still unclear. SDG may associate with head trauma and is considered as a possible complication of DC [5]. The average time from DC to the onset of contralateral SDG was 14 days in Yang et al.'s series of works. The pathomechanism by which SDG develops is unknown [17, 25, 26]. Several possible mechanisms could be responsible for the development of contralateral SDG following DC. One commonly accepted theory is that rapid reduction in ICP as well as outward herniation after decompression may produce a pressure gradient between the two hemispheres which lead to accumulation of CSF and enlargement of the contralateral subdural space, especially when, initially, there was a possible rupture in the arachnoid layer after head trauma [13, 27]. Other factors, such as rupture of the arachnoid layer, result in the formation of a one-way valve that promotes CSF leakage and fluid accumulation in the subdural space, which, in contrast, prevents reabsorption of CSF [13]. This appears compatible with respect to the etiology of post-traumatic SDG. Other important factors of equal importance include shrinkage of the brain due to intraoperative tissue retraction and CSF drainage with subsequent inability of the brain to regain its normal shape [13]. Under such circumstances, a disturbance of normal CSF absorption may enhance the risk of CSF leakage through the torn portion of the arachnoid membrane.

Although the evolution of SDG is widely discussed, its consequences are variable. Most SDGs are clinically silent and asymptomatic [8]. In general, the overall course of SDG can rarely be associated with significant mass effect. Only few patients with SDGs present with stupor or coma. Management of SDGs includes conservative management with close observation and surgical treatment. Conservative management is recommended when SDG is small, no manifest brain shift and asymptomatic. In contrast, if radiological evidence exists including obvious compression of a cerebral ventricle and cistern and midline shift of the midline and deterioration of clinical presentation due to progressed SDGs, surgery should be performed as soon as possible. Specifically, it has been suggested for surgery that SDG larger than 7 mm with accompanying symptoms, or asymptomatic but larger than 10 mm [18]. For asymptomatic SDGs with a large quantity of fluid, we tend to treat them with simultaneous burr hole evacuation and cranioplasty. Even without burr hole evacuation, many small ones simply resolve spontaneously following replacement cranioplasty. Rarely, they should need shunting procedures for these asymptomatic SDGs.

In comparison, contralateral SDGs are more likely to be symptomatic than unilateral ones. Contralateral SDGs are usually combined with external cerebral herniation. Expansion of the brain with external cerebral herniation through the skull defects is often observed in the early period after DCs [5]. Contralateral SDGs need more aggressive treatment because of their tendency to cause midline shift and neurologic deterioration [12]. Furthermore, evacuation via burr hole for contralateral SDGs is likely to carry the risk of failure. SDG may be resistant to simple burr hole drainage technique, especially when the SDG is combined with a contralateral large skull defect, with possible pressure gradient between the hemispheres that would contribute to reaccumulation of CSF [14]. Although a burr hole drainage is simple and eliminates the potential complications of an intracranial shunt, this may be offset due to contralateral SDG recurrences needing further revision surgeries, which could be hazardous to the patient. A study by Wang and associates [27] tried to evacuate contralateral SDG in 6 patients with neurological deficits, using the burr hole drainage technique. One patient received subsequent SP shunting due to recurrence of the SDG.

In the current study, the proposed surgical methods, SP shunting plus cranioplasty, were indicated in patients who suffered from acute conscious disturbance or remarked neurological deficits caused by the external cerebral compromise from contralateral SDG. For such critical patients, immediate surgical decompression is pivotal and hence these patients cannot afford any revision surgeries because of inadequate initial decompression in burr hole evacuation. Moreover, it is likely to be criticized that our encouraging results are due to the reason that our recommendation of shunting appears to be “an overkill.” However, for the sake of saving critical lives, such effective shunting method offers an option for treatment of contralateral SDGs with mass effects.

It is suggested that SP shunt could be the best choice in surgical treatment when SDG has a large quantity of fluid and increases in size progressively [28]. The current study focuses on patients with contralateral SDGs, which progressed rapidly in size and caused midline shift and neurologic deterioration. Because of the potential failure in burr hole evacuation for contralateral SDGs and the need for prompt rescue surgery in our patients with neurological deficits, we simultaneously performed cranioplasty thoroughly resolving the outward herniation, and the resultant pressure gradient developed by the cranioplasty facilitates the drainage effect of hygroma by the contralateral SP shunt. Our data demonstrated successful treatment of the contralateral SDGs developed after DCs, by performing SP shunt and cranioplasty simultaneously. No patient in our study experienced reaccumulation of subdural fluid after going through our suggested surgical procedure. Although simultaneous cranioplasty and SP shunt have been described in few case reports [14], our study provides a series of clinical experiences to prove the practical value.

Hydrocephalus might develop after SP shunting for SDG. SP shunting plus cranioplasty thoroughly resolves SDG collection and might facilitate narrowing and adhesions in the subdural and subarachnoid spaces, leading to hydrocephalus by deterioration in CSF circulation [29]. In the current study, 2 patients develop symptomatic hydrocephalus presenting with drowsiness and general weakness after 65 days following cranioplasty plus SP shunting surgery. Double gradient shunting procedure, VP shunt following SP shunt, is used to manipulate the CSF circulation disturbance. In the two patients with hydrocephalus after SP shunt plus cranioplasty, we performed a programmable VP shunt to restore and reorganize the brain tissue into a near normal anatomy. To prevent the recurrence of SDG following double gradient shunting, programmable VP shunt should be set with high pressure initially and may be adjusted to lower pressure and monitored by clinical manifestation.

Although the described technique is simple, practical, and efficient, the study included a relatively small number of patients. Future large-scale studies are warranted to examine its practicability in TBI patients sustained contralateral SDGS following DCs.

## 5. Conclusion

We effectively resolved contralateral SDGs using one-stage, simultaneous cranioplasty and contralateral SP shunting. Our excellent results included the completely resolved SDGs in radiological studies and significant recovery in neurological deficits caused by SDGs. This surgical combination technique seems feasible and practical and gives excellent functional outcomes with few complications in TBI patients undergoing contralateral SDGs following DCs.
